# Epidemiology of Orthopedic Fractures and Other Injuries among Inpatients Admitted due to Traffic Accidents: A 10-Year Nationwide Survey in Taiwan

**DOI:** 10.1155/2014/637872

**Published:** 2014-02-05

**Authors:** Ren-Hao Pan, Nien-Tzu Chang, Dachen Chu, Kuo-Fang Hsu, Yuan-Nian Hsu, Jin-Chyr Hsu, Lin-Yu Tseng, Nan-Ping Yang

**Affiliations:** ^1^Department of Emergency Medicine, Taoyuan General Hospital, Ministry of Health & Welfare, Taoyuan 33004, Taiwan; ^2^Department of Orthopedic Surgery, Taoyuan General Hospital, Ministry of Health & Welfare, Taoyuan 33004, Taiwan; ^3^Department of Computer Science and Engineering, National Chung Hsing University, Taichung 40227, Taiwan; ^4^Department of Nursing, College of Medicine, National Taiwan University, Taipei 10617, Taiwan; ^5^Institute of Public Health, National Yang-Ming University, Taipei 11221, Taiwan; ^6^Department of Neurologic Surgery, Taipei City Hospital, Taipei 10341, Taiwan; ^7^Department of Computer Science and Communication Engineering, Providence University, Taichung 43301, Taiwan

## Abstract

To investigate the major injury patterns associated with traffic accidents and evaluate the risk factors of the main injury, a survey of Taiwan's national insurance admission data between 2002 and 2011 was performed. The incidence of traffic-accidents-related hospitalization was between 9.17% and 11.54% and the average mortality rate of the inpatients admitted due to traffic accidents was 0.68%. Of all inpatients due to road traffic accidents in Taiwan, orthopedic fractures were the most common injuries that accounted for 29.36% of them. There were a total of 391,197 cases of three orthopedic fracture groups that were divided into (1) fracture of upper limb, (2) fracture of lower limb, and (3) fracture of spine and trunk. An increase in national medical cost used for inpatients with orthopedic fractures was noted and ranged from US$ 45.6 million to US$ 86 million annually. These orthopedic fracture patterns were frequently associated with other injuries especially head injuries (ranged from 14% to 26%). A significant relation to male gender, older age, low income, and admission to high-level hospital to the observed fracture patterns was observed.

## 1. Introduction

Road traffic injures (RTIs) are responsible for a substantial proportion of deaths and injuries and are responsible for more years of life lost than most human diseases. Human behavior factors, vehicle factors, and road factors contribute to the causation of road traffic crushes [1]. Although the numbers of lives lost in road crushes in high-income countries indicate a downward trend in recent decades, for most of the world's population, the burden of road-traffic injury in terms of societal and economic costs is rising substantially [2]. The distribution of road traffic deaths by road user group varies dramatically across epidemiological WHO subregions and also varies across low-income, middle-income, and high-income countries. For example, 45% of road traffic fatalities in low-income countries are among pedestrians, whereas an estimated 29% in middle-income and 18% in high-income countries are among pedestrians [3]. Global efforts to reduce road traffic injuries may be facilitated. For example, motorcycle helmets were found to reduce the risk of head injury and from five well-conducted studies the risk reduction is estimated to be 72% (odds ratio (OR): 0.28, 95% confidence interval (CI): 0.23–0.35) although there was some evidence that the effect of helmets on mortality is modified by speed [4].

There is a dearth of information on injury patterns that could be used to prioritize injury prevention measures. For example, hospital discharge data from eight European countries, including 10,341 pedestrians sustaining 19,424 injuries, have been used and fractures (51.1%, 95% CI: 50.3–51.8) and internal injuries (21.3%, 95% CI: 20.7–21.9) are the most frequently found in the data [5]. From the viewpoint of preventive medicine, a comprehensive survey of nationwide traumatic epidemiology, especially related to traffic accidents, should be necessary for Taiwan's health authority to redistribute medical resources to the major injuries. The present study, based on the Taiwan's national admission data, is aimed to (1) investigate the occurrence of injuries associated with traffic accidents, (2) find the major distributions of injury patterns, and (3) evaluate the risk factors of the main injury.

## 2. Materials and Methods

### 2.1. Data Sources

From March 1, 1995 to 2011, 23.199 million citizens were enrolled in the single-payer national health insurance (NHI) program and reached 99.88% of the total population of Taiwan. This universal national health insurance [6], jointly financed by payroll taxes, subsidies, and individual premiums, commenced in Taiwan, has consistently received a 70 percent public satisfaction rate. The patient data analyzed in this research were obtained from the National health Insurance Research Database (NHIRD), which is maintained by the Bureau of National Health Insurance (BNHI) and the National Health Research Institute (NHRI) for research purpose. The academic databank of NHIRD included various subdatasets, for example, inpatient expenditures by admissions (DD), details of inpatient orders (DO), ambulatory care expenditures by visits (CD), and details of ambulatory care orders (OO). In this study, the DD dataset was used for further exploration.

### 2.2. Data Protection and Permission

For the personal information protection, all datasets were already passed the scrambling procedure before being sent to the NHRI and, consequently, the dataset of NHIRD was not used to identify the patient's personal information. Essentially, it is unable to restore the original data when using this database. Moreover, the researcher who attempts to use the NHIRD is necessary to declare without intention to obtain information which could potentially violate the privacy of patients or care providers. This study was approved by the Institutional Review Board (IRB) of Taoyuan General Hospital, which has been certified by the Department of Health, Taiwan (IRB Approval number: TYGH101049), and the protocol was evaluated by the NHRI, who gave their agreement to the planned analysis of the NHIRD (Agreement number: NHIRD-101-566).

### 2.3. Data Selection and Definition

In this study, we focus on the data obtained from inpatients due to road traffic accidents in one-decade period between January 1, 2002, and December 31, 2011. All patients were eligible for this study when the entry of admissions (DD) dataset of NHIRD marked traffic event TRAC_EVEN, the total number of selection patient was 654,571 people, and then 653,386 patients remained from the prefiltered of missing data. In order to investigate the contribution of the categorical diagnoses associated with injuries, the International Classification of Diseases, 9th Revision, Clinical Modification (ICD-9-CM) diagnosis codes and ICD-9-CM treatment codes were evaluated. Thus, 20 groups of diagnoses were defined (in Table 4): the fracture of skull, intracranial injury (800–804 and 850–854 series), the fracture of spine and trunk (805–809 series), the fracture of upper limb (810–819 series), the fracture of lower limb (820–829 series), the dislocation (830–839 series), the sprains and strains of joints and adjacent muscles (840–848 series), the internal injury of chest (860–862 series), internal injury of abdomen and pelvis (863–869 series), the open wound of head, neck, and trunk (870–879 series), open wound of upper limb (880–887 series), the open wound of lower limb (890–897 series), the injury to blood vessels (900–904 series), the late effects of injuries and other external causes (905–909 series), the superficial injury (910–919 series), the contusion with intact skin surface (920–924 series), the crush injuries (925–929 series), the effects of foreign body entering through orifice (930–939 series), the burns (940–949 series), the injury to nerves and spinal cord (950–957 series), and the certain traumatic complications and unspecified injuries (958–959 series).

### 2.4. Statistics

In the analysis of this study, the comparison of baseline characteristics by descriptive statistics, represented by the numbers of cases, gender, percentages, and means with standard deviation (SD), and incidence was made. The individual and cluster differences of orthopedic fractures were analyzed by the mix model with 95% confidence intervals (95% CIs). To evaluate the risk factors of specific orthopedic fracture pattern, multiple logistic regression method was used. Multilevel analysis was used as an analytical strategy by allowing the examination of group-level and individual-level factors [7]. For the distributions of their major combined injuries, significance was set at *P* = 0.05. All statistical analyses were performed using the Statistical Package for Social Sciences for Windows (SPSS for Windows 18.0).

## 3. Results

**Figure 1:**
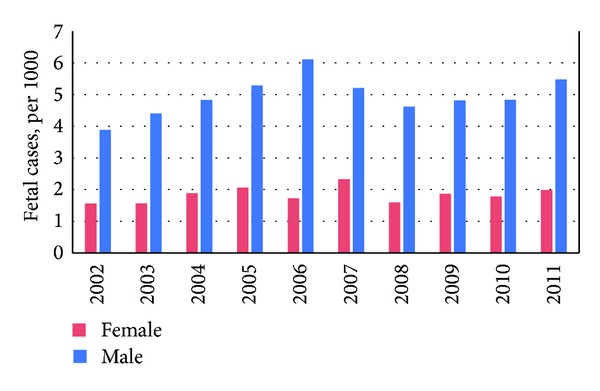
Mortality rates of both genders among the traffic accidents-related inpatients in Taiwan, 2002–2011.

The basic characteristics of the enrolled subjects admitted due to the traffic accidents between 2002 and 2011 were calculated.  Table 1 shows the trends of incidence of traffic accidents-related hospitalization: 9.21% in 2002, 9.17% in 2003, and 10.21%, 10.02%, 10.10%, 9.84%, 9.87%, 9.66%, 10.39%, and 11.54% during 2004–2011, respectively. In general, the gender proportions were male 59.4% and female 40.5%. The averaged distributions of age stratum in the studied population were 0–17 years (7.04%), followed by 18–29 years (29.07%), 30–49 years (31.28%), 50–64 years (24.17%), and the level for more than 65 years (16.51%).  Figure 1 shows the trend of mortality rate between both genders during the period of 2002–2011. Estimated total mortality cases were 4,452 (female: 1,205 cases, male: 3,247 cases), the average mortality rate was 0.68% of the inpatients admitted due to traffic accidents (female: 0.18%, male: 0.50%). Compared to the mortality rate in both gender, the average of incidence ratio (IR) between females and males was 1 : 2.7.

The 653,386 inpatients populations of traffic accident-related were classified into 20 groups of diagnosis categories (shown in Table 4) and ranked by the summed cases of each group; the top 10 groups with high incidence rate were listed. The most injury patterns were the fracture of skull, intracranial injury (17.93%), contusion with intact skin surface (12.58%), the fracture of upper limb (12.22%), the fracture of lower limb (12.22%), the superficial injury (10.51%), open wound of head, neck, and trunk (8.94%), fracture of spine and trunk (6.10%), open wound of lower limb (4.79%), certain traumatic complications and unspecified injuries (3.73%), and open wound of upper limb (2.42%) (shown in Table 2). In total, 391,197 cases of three orthopedic fracture groups were divided into (1) fracture of upper limb, (2) fracture of lower limb, and (3) fracture of spine and trunk, which could be integrated to be the major injury (orthopedic fracture) that accounted for 29.36% of all the traffic incidents-related inpatients. Their medical utilizations, including direct medical cost and length of stay (LOS), were calculated. Focused on the three fractured cases, the mean cost was US$1260, US$1905, and US$1611 and the mean LOS were 5.9 days, 8.7 days, and 8.7 days for the upper limbs fractures, lower limbs fractures, and the spine/trunk fractures, respectively (still shown in Table 2).

**Figure 2:**
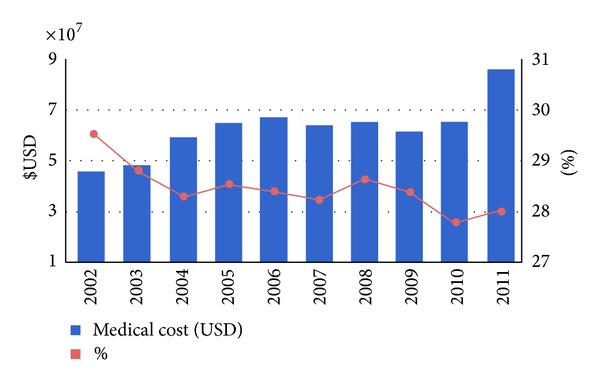
Trends of direct medical burden of the orthopedic fractures among all the inpatient population in Taiwan, 2002–2011.

**Figure 3:**
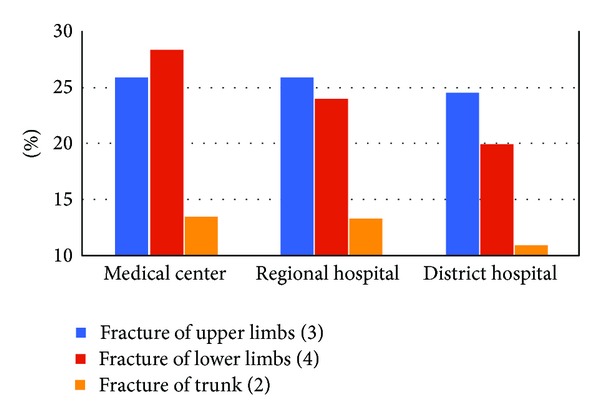
Percentage of three orthopedic fracture patterns of all the traffic accidents-related inpatients treated among three different level hospitals in Taiwan, 2002–2011.

Figure 2 estimated the direct medical burden of the orthopedic fractures among all the admitted population; the trend of analysis showed an increase in summed medical cost of three orthopedic fracture subgroups during the period of 2002–2011 (ranged from US$ 45.6 million to US$ 86 million). Although an increased total medical cost was noted, the percentage of cost spent on treatments of orthopedic fractures compared to cost spent on treatments of all traffic incidents-related injuries was decreased from 29.5% to 28%.  Figure 3 showed the distributions of three orthopedic fracture patterns treated among three-leveled hospitals in Taiwan, including medical center, regional hospital, and district hospital. Higher-leveled hospitals received higher proportions of three categorical orthopedic fractures, and the medical centers had to treat more lower-limb fractures among their traffic incidents-related inpatients.

The risk factors of the three categorical orthopedic fractures were evaluated and shown in Table 3. Male was noted to be a significant risk factor in fracture of lower limbs (odds ratio (OR) 1.003, and 95% Confidence Interval (CI) 1.001–1.005) and fracture of trunk (OR 1.006, 95% CI 1.004–1.008). Compared with the age stratum of 18–29 years, increased ages were almost related to increased ORs of upper-limb and spine/trunk fractures but the younger population (less than 18 years old) preferred to get a lower-limb fracture (OR 1.055, 95% CI 1.050–1.059). Compared to the district hospitals, higher-leveled hospitals (regional hospitals) and the highest-leveled hospitals (medical centers) had an increased tendency to treat the orthopedic fracture cases, especially for the lower-limb or spine/trunk fractures. Otherwise, low socioecological level population was also noted to have more opportunities to get a lower-limb fracture or a spine/trunk fracture (ORs were 1.019 (95% CI 1.012–1.026) and 1.008 (95% CI 1.003–1.014), resp.). The major combined injuries of three categorical fractures were still evaluated. Head injury (recorded as fracture of skull, intracranial injury) was the most combined injury (incidence ranged from 14% to 26%), followed by contusion with intact skin surface (incidence ranged from 12% to 25%) and superficial injuries (incidence ranged from 13% to 18%).

## 4. Discussion

An international evaluation of the Global Burden of Diseases, Injuries, and Risk Factors Study 2010 (GBD 2010), identifying all available data on causes of death for 187 countries from 1980 to 2010, showed that the fraction of global deaths due to injuries was marginally higher in 2010 (9.6%) compared with two decades earlier (8.8%). This was driven by a 46% rise in worldwide deaths due to road traffic accidents and a rise in deaths from falls [8]. Because of this significant burden, the primary purpose of this study was to explore the incidences of injuries associated with traffic accidents and their modifiable risk factors to promote the advance of health and injury-prevention policies in Taiwan.

In this present study, annual road traffic injury incidences rate in recent ten years was from 9.17% to 11.54%; the highest was in 2011. These high incidences were similar to those reported in the Vorko-Jović (2006) study, which focused on urban road traffic accidents in Croatia [9]. Another similar result was also found in an America study [10], which also reported the gender difference that males are much more likely to get injury in a road traffic crash than females, especially among adults and the elderly. Our study was consistent with previous studies.  Table 1 and Figure 1 demonstrate that males are much more likely to be killed in a road traffic injury than females.

Head and spine injuries were most common among front and rear vehicle occupants and drivers [11]. Among motorcycle riders admitted to the hospital, the most common head injuries are concussions, followed by brain injuries or hemorrhage, facial fractures, and skull fractures. In Taiwan, 18% of the inpatients who suffered from head injuries were estimated in the present study. Around 7.6% to 75.1% of motorcycle riders got head injuries without helmets; oppositely, the incidence of head injuries in motorcycle riders who wear helmets is 3.4% to 40.6% [12]. These highly differences of injury incidence between helmeted or nonhelmeted riders were similar to those reported in a meta-analysis study. Motorcycle helmets were estimated to reduce 72% risk of any kinds of head injury (odds ratio (OR): 0.28, 95% confidence interval (CI): 0.23–0.35) [4]. That is another issue worthy to be investigated in Taiwan.

Head injuries are the leading cause of death in motorcycle accidents, even in helmeted riders. For instance, in the US, 53% of motorcycle deaths were a result of head injuries [12]. Incidence of head injuries caused by a rotational acceleration has been pointed out in the literature, but they underestimate the number of pedestrian fatal victims in eight European countries and in San Francisco study [5, 13]. A previous study in the United Arab Emirates reported that road traffic victims are predominantly male (89%), pedestrians (88%). In UAE study, the Trauma Registry of Al Ain city was collected over 3 years, and showed that there were 1070 patients, mainly from non-Arabic speaking expatriates, low-income countries. Overall mortality was 4%; pedestrians accounted for 61% of deaths. Head injury was the major factor affecting hospitalization and mortality [11].

In the USA, 5,838 admissions of an academic Level I trauma center registered over 10 years were reviewed and showed that there were 1,136 patients (19.4%) 14 years old or less, 3,741 (64.1%) who were 15 to 55 years old, 420 (7.2%) 56 to 65 years old, and 541 (9.3%) older than 65 years. Overall mortality was 7.7% and ranged from 3.2% in the age group of 14 years or less to 25.1% in patients over 65 years [14]. In the USA, another National Trauma Databank study during a 5-year period including 12,429 admissions revealed that there were 4,095 patients (32.9%) ≤14 years, 3,806 (30.7%) 15 to 35 years old, 3,413 (27.5%) 36 to 55 years old, 688 (5.5%) 56 to 65 years old, and 427 (3.4%) >65 years old, The overall mortality was 3.7% and ranged from 2.4% in the age stratum of ≤14 years to 12.2% in the stratum of >65 years [15]. In the present study, the traffic incidents-related mortality rate among those admitted populations was noted as a significant gender difference in Taiwan (in average, 0.18% for the female and 0.50% for the male).

A cross-sectional study in India showed that fractures were the commonest injury among the victims of nonfatal road traffic accidents, and majority of the victims were in the age group of 18–37 years [16]. A road trauma analysis based on Data of the Trauma Registry in the United Arab Emirates showed that injuries of the extremities and head were frequent among pedestrians, motorcyclists, and bicyclists; the mean hospitalization was 9.7 days and overall mortality was 4% [11].

In China, the data of 2213 inpatients with traffic trauma showed that fracture of extremities (53.3%) occurred most often, craniocerebral trauma (19.4%) next, then followed in turn by thoracoabdominal visceral injury (6.56%), spine fracture (5.37%), fracture of ribs (4.88%), and pelvic fracture (4.18%) [17]. In Africa, a retrospective analysis of nonfatal road traffic crash victims still showed that the commonest injuries were fractures (69.0%) with the tibia/fibula being the most fractured bones (30.3%). Age group of 15–44 years was the most affected (81.9%) [18]. In the present, fractures of upper limb, lower limb, and spine and trunk account for about 30% of the inpatients caused by traffic incidents in Taiwan.

The USA National Trauma Databank study of 12,429 admissions showed that bicycle-related injuries involving motor vehicles are associated with a high incidence of head injuries and extremity fractures. Age plays a critical role in the severity and anatomic distribution of injuries sustained, with a stepwise increase in mortality with increasing age [15]. In Pakistan, of the 132,504 victims of road traffic crashes (RTCs), there were 67% males and 65% aged 16–35 years, and minor injuries (65%) and fractures (25%) were the most reported [19]. Another hospital-based study of 450 cases admitted due to traffic accidents in India revealed that the commonest type of injury was fracture (49.33%) and the most common site of fracture was lower limb (48.2%), and several risk factors such as age, sex, type of vehicle, use of alcohol, absence of driving license, nonuse of helmets, and casual attitude are associated with increased occurrence of road traffic accidents [20]. In the present study, gender, age, and socioeconomic level were significant risk factors of the most orthopedic fractures among traffic incidents-related inpatients in Taiwan. Otherwise, different hospital level receiving these orthopedic fractured cases was another cluster factor found in the present study.

The study of national estimates of motor vehicle crash- (MVC-) related hospitalization and associated use of health care resources among patients of 20 years old and younger in 3438 hospitals in 36 USA states revealed that mean (SD) hospital charges and lengths of stay (LOS) were $33,440 ($55,113) and 4.8 (7.7) days, respectively. Older age, being male, urban hospital location, mortality during hospitalization, higher injury severity, and longer LOS were significantly associated with higher total charges [21]. Another study based on the Iranian National Trauma Registry Database (INTRD), including data from 14 general hospitals in eight major cities in Iran, enrolled 8,356 patients with road traffic injuries (RTIs) admitted to the hospitals and showed that the mean hospital charges for the patients were US$128 ± US$210 and the mean LOS for the patients was 6.8 ± 8.0 days. Older age, being a bicycle rider, higher injury severity, and longer LOS were associated with higher hospital charges [22]. Compared to the two above studies, the direct medical cost and LOS for the traffic incidents-related hospitalization in Taiwan would be reasonable and accessible to the people. Furthermore, our study demonstrates that integrating all road users and pedestrian patients with hospital discharge data provides better estimates of the incidence of injury and more comprehensive information about injury type than other local hospital-based ED reports [23].

## 5. Conclusion

Orthopedic fractures were the most common injuries among inpatients due to road traffic accidents in Taiwan from 2002 to 2011. They were frequently associated with other injuries especially head injuries. A significant relation to male gender, older age, low income, and admission to high-level hospital to the observed fracture patterns was observed.

## Figures and Tables

**Table 1:** Basic characteristics of the enrolled population, 2002–2011.

Year	Male		Female		Total	Incidence*	Age-stratum distributions of the enrolled subjects (%)				
	No.	(%)	No.	(%)	No.		<18 y/o	18–29 y/o	30–49 y/o	50–64 y/o	≥65 y/o
2002	35880	59.24	24265	40.06	60145	9.21	8.72	29.40	29.90	18.85	13.13
2003	35966	59.49	23971	39.65	59937	9.17	8.10	28.66	29.88	19.76	13.28
2004	40055	59.83	26646	39.80	66701	10.21	8.06	31.80	32.66	22.69	14.81
2005	39366	60.15	26075	39.85	65441	10.02	8.16	30.65	31.23	22.77	15.28
2006	39777	60.29	26197	39.71	65974	10.10	7.44	29.67	31.66	23.95	16.25
2007	38236	59.45	26079	40.55	64315	9.84	6.77	28.50	30.45	24.08	16.66
2008	38090	59.07	26393	40.93	64483	9.87	6.36	27.38	30.86	24.82	17.33
2009	36747	58.21	26386	41.79	63133	9.66	5.53	26.06	30.20	25.48	17.42
2010	39665	58.45	28196	41.55	67861	10.39	5.60	28.00	31.36	27.75	19.40
2011	44172	58.59	31224	41.41	75396	11.54	5.66	30.56	34.61	31.59	21.49

*Incidence of traffic accidents-related admissions among all the inpatients recorded in the national health insurance data (1/100).

**Table 2:** The most 50% of admitted diagnoses related to traffic incidents and their effects on the medical utilizations.

Categorical diagnoses (grouping in Table 4)	Summed cases, 2002–2011			Incidence*	Length of stay (days)		Medical costs (US$)	
	No.	Male (%)	Female (%)		mean	SD	mean	SD
Fracture of skull, intracranial injury (1)	238659	59.81%	40.19%	17.93%	7.04	8.27	1,492.67	2,793.70
Contusion with intact skin surface (15)	167465	54.81%	45.19%	12.58%	4.95	4.22	668.61	994.00
**Fracture of upper limb (3)**	162665	58.75%	41.25%	**12.22%**	5.90	5.87	1,259.51	1,736.84
**Fracture of lower limb (4)**	147348	60.00%	40.00%	**11.07%**	8.72	7.71	1,904.96	2,533.22
Superficial injury (14)	139886	61.18%	38.82%	10.51%	5.03	4.29	732.91	953.86
Open wound of head, neck, and trunk (9)	119012	66.92%	33.08%	8.94%	5.89	5.75	1,051.83	1,613.72
**Fracture of spine and trunk (2)**	81184	59.26%	40.74%	**6.10%**	8.66	8.76	1,611.04	2,818.54
Open wound of lower limb (11)	63826	60.13%	39.87%	4.79%	7.29	7.15	1,150.49	1,713.59
Certain traumatic complications and unspecified injuries (20)	49623	60.86%	39.14%	3.73%	7.61	8.93	1,639.47	3,126.97
Open wound of upper limb (10)	32203	71.70%	28.30%	2.42%	5.76	5.43	988.17	1,410.63

*The percentage of admitted diagnosis among the 20 categorical diagnoses (see Table 4) associated with injuries. The exchange rate US dollar (USD) to Taiwan dollar (TWD) is 1 : 30.

**Table 3:** Individual and cluster differences of orthopedic fractures analyzed by the multiple logistic regression and distributions of their major combined injuries.

Independent factors	Fractures of upper limbs (3)	Fracture of lower limbs (4)	Fracture of trunk (2)
	AOR (95% CI)	AOR (95% CI)	AOR (95% CI)
Gender			
Female	1.0	1.0	1.0
Male	0.999 (0.997, 1.001)	**1.003 (1.001, 1.005)**	**1.006 (1.004, 1.008)**
Age stratum			
<18 y/o	**0.982 ( 0.978, 0.987)**	**1.055 (1.050, 1.059)**	**0.967 (0.963, 0.970)**
18–29 y/o	1.0	1.0	1.0
30–49 y/o	**1.037 (1.034, 1.040)**	**0.988 (0.985, 0.991)**	**1.070 (1.067, 1.072)**
50–64 y/o	**1.061 (1.057, 1.064)**	0.998 (0.995, 1.001)	**1.130 (1.127, 1.133)**
≥65 y/o	0.999 (0.995, 1.002)	**1.047 (1.043, 1.050)**	**1.135 (1.132, 1.139)**
Socioeconomic level			
Normal population	1.0	1.0	1.0
Low-income population	**0.991 (0.984, 0.998)**	**1.019 (1.012, 1.026)**	**1.008 (1.003, 1.014)**
Hospital level			
Medical center	1.051 (0.964, 1.146)	**1.107 (1.035, 1.183)**	**1.053 (1.026, 1.082)**
Regional hospital	**1.059 (1.016, 1.104)**	**1.070 (1.036, 1.105)**	**1.035 (1.022, 1.049)**
District hospital	1.0	1.0	1.0

Combined injuries	Incidence (%), 95% CI	Incidence (%), 95% CI	Incidence (%), 95% CI

Fracture of skull, intracranial injury (1)	19% (18.9, 19.1)	14% (13.9, 14.1)	26% (25.8, 26.2)
Contusion with intact skin surface (15)	16% (15.9, 16.1)	12% (11.9, 12.1)	25% (24.8, 25.2)
Superficial injury (14)	17% (16.9, 17.1)	13% (12.9, 13.1)	18% (17.8, 18.2)
Open wound of head, neck, and trunk (9)	11% (10.9, 11.1)	10% (9.9, 10.1)	12% (11.9, 12.1)
Open wound of lower limb (11)	5% (4.9, 5.1)	10% (9.9, 10.1)	5% (4.9, 5.1)
Certain traumatic complications and unspecified injuries (20)	6% (5.9, 6.1)	6% (5.9, 6.1)	9% (8.8, 9.2)
Open wound of upper limb (10)	5% (4.9, 5.1)	3% (2.9, 3.1)	3% (2.9, 3.1)

AOR: adjusted odds ratio; 95% CI: 95% confidence interval.

**Table 4:** 20 groups of categorical diagnoses associated with injuries.

Group	ICD-9-CM codes	Descriptions
1	800-804, 850-854	Fracture of skull, intracranial injury
2	805-809	Fracture of spine and trunk
3	810-819	Fracture of upper limb
4	820-829	Fracture of lower limb
5	830-839	Dislocation
6	840-848	Sprains and strains of joints and adjacent muscles
7	860-862	Internal injury of chest
8	863-869	Internal injury of abdomen and pelvis
9	870-879	Open wound of head, neck, and trunk
10	880-887	Open wound of upper limb
11	890-897	Open wound of lower limb
12	900-904	Injury to blood vessels
13	905-909	Late effects of injuries and other external causes
14	910-919	Superficial injury
15	920-924	Contusion with intact skin surface
16	925-929	Crush injuries
17	930-939	Effects of foreign body entering through orifice
18	940-949	Burns
19	950-957	Injury to nerves and spinal cord
20	958-959	Certain traumatic complications and unspecified injuries
